# Editorial: Tertiary Lymphoid Structures: From Basic Biology to Translational Impact in Cancer

**DOI:** 10.3389/fimmu.2022.870862

**Published:** 2022-03-16

**Authors:** Catherine Sautès-Fridman, Anna Dimberg, Vivek Verma

**Affiliations:** ^1^ Centre de Recherche des Cordeliers, INSERM, Université de Paris, Sorbonne Université, Equipe inflammation, complément et cancer, Paris, France; ^2^ Equipe labellisée Ligue contre le Cancer, INSERM, Université de Paris, Sorbonne Université, Equipe Inflammation, Complément et Cancer, Paris, France; ^3^ Department of Immunology, Genetics and Pathology, Rudbeck Laboratory, Science for Life Laboratory, Uppsala University, Uppsala, Sweden; ^4^ The Hormel Institute, University of Minnesota, Austin, MN, United States

**Keywords:** biomarker, development, tumor microenvironment, *in situ* immune response activation, *in situ* generation method

Tertiary lymphoid structures (TLSs) are organized lymphoid aggregates developing in inflamed tissues upon infection, auto-immune reactions, and in tumors. As potential biomarkers of response to antibodies against immune checkpoints, TLSs have recently become prominent, highlighting the need to increase basic knowledge of these structures. This Research Topic presents 14 articles covering TLS heterogeneity and relationships with tumor mutational burden (TMB) as well as strategies for their induction in mouse models and in artificial scaffolds.

## The Early Events Triggering TLS Formation

The question regarding the early events that lead to TLS formation has been addressed in mouse models. Johansson-Percival and Ganss highlight that development of lymphoid tissue involves a crosstalk between Podoplanin^+^ICAM^+^/αSMA^+^ stromal cells expressing LTβR and TNFR [LT organizer cells (LTo)] and immune cells including macrophages, dendritic cells (DCs), and T and B cells [LT inducer cells (LTi)]. This interaction sustains the production of CCL19/21/CXCL13 by stromal cells through production of TNFα, LTα and β, LIGHT, IL1β, and CCL21. They discuss the LTi role of each of the immune cell types to support mature TLS formation. They hypothesize that CCL19-secreting cancer-associated fibroblasts, TNFα-secreting macrophages, and LTβ-secreting DCs could be the originators of TLSs in human cancers. Filderman et al. cite the role of interferon (IFN) and IL1 family members (ILF9/IL36γ), in association with activation of T cell-mediated anti-tumor functions for TLS induction.

TLSs are surrounded by high endothelial venules (HEV). Filderman et al. provide a comprehensive review on the chemokines and cytokines involved in HEV formation and TLS induction from studies in mouse models. TNFR- over LTβR-mediated signaling is dominant for HEV and TLS neogenesis (Filderman et al.), both pathways being necessary for acquisition of a fully mature HEV phenotype (Vella et al.). HEVs can be modulated by immune cells, DCs favor their formation and Tregs suppress it. Vella et al. provide evidence suggesting that altered HEVs could allow formation of a pre-metastatic niche permissive for tumor cells, and allow tumor cell intravasation into the bloodstream.

## TLSs: A Set of Structures With Distinct Cellular Composition and Functions

By analogy with secondary lymphoid organs (SLOs), three classes of TLSs are being considered (1): primary follicle-like TLSs with CD21^+^ follicular dendritic cells (FDCs) (2), mature TLSs with CD23+CD21+ FDC within germinal centers (GC) and lymphoid aggregates of T and B cells, and (3) so-called, although not demonstrated, “early-TLSs” or “immature TLSs”.

One of the current key issues is to deeply characterize these distinct TLSs and understand their relationships with the immune infiltrate and the surrounding tissue. Two papers address these questions in urothelial cancers (UCs). UCs develop below inflamed areas of the bladder and can become invasive in the muscle. By analyzing 40 tumor samples of muscle invasive bladder cancer patients (MIBC), Pagliarulo et al. show that immature TLSs have an increased proportion of T cells and reduced proportion of total B cells when compared to mature TLSs. Presence of mature TLSs correlates with higher lymphocytic infiltration in many cancer types. Analyzing the TME, Pagliarulo et al. find that the majority of TLS-high tumors show B and T cell co-infiltration harboring naïve (PD1-TCF7+) and progenitor-like (PD1+TCF7+) CD8 T cells and activated B cells (PD1+), whereas only a minority of TLS-low tumors present this type of infiltration. These differences are independent of TLS maturation stages. Pagliarulo et al. therefore suggest a role of B cells and CD8 T cell interactions sustaining the presence of PD1+ TCF1+ T cells in the TME. Trüb and Zippelius also discuss the mechanisms underlying the potential crosstalk between B cells and CD8 T cells, either direct as suggested by Pagliarulo et al. or through DCs or CD4 T cells, leading to optimal cancer immunosurveillance. Which molecules are involved in a potential direct B cell-CD8 T cell crosstalk need to be further explored.

Van Dijk et al. compare TLSs in cystectomy specimens and in superficial transurethral resection (TUR) biopsies of 31 UC patients. TLSs located in TUR biopsies display higher numbers of CD4 T cells, a higher fraction of early TLS, and lower germinal center (GC+) TLS than submucosal ones. TUR specimens contain superficial tissue that is highly exposed to inflammation stimuli, urinary toxins, and microbial pathogens. The Van Dijk et al. report is reminiscent of studies showing that the early hepatic lesions that precede transformation to hepatocellular carcinoma (HCC) also display immature TLS and show elevated expression of immune inhibitory molecules that may favor immune evasion (1). Further experiments are needed to investigate the prognostic impact of these distinct entities and the antigens recognized. In the same line, Werner et al. investigate TLS composition and location by seven-color multiplex staining of whole tissue sections in 48 patients with primary cutaneous melanoma and 39 distant/late metastases. Whereas only early TLSs were found in one third of primary tumors, half of the metastases contained secondary follicle-like TLSs most of which were located in the extratumoral compartment within 1 mm distance to the invasive tumor. Most of these TLSs lack BCl6+ lymphatic cells and canonic GC polarity. This paper shows that the “mature TLS” stage defined using CD21+CD23+FDC may, depending on the tumor site, include heterogeneous phenotypes with variable BCl6 expression levels in B cells, likely reflecting the strength of B cell signaling and of T cell help, and thus the fate of B cell differentiation into antibody secreting cells. Other types of TLS heterogeneity could influence the impact of mature TLS on prognosis. Johansson-Percival and Ganss mention that TLSs with high densities of M2 macrophages and T helper cells expressing GATA3, a master regulator of Th2 differentiation, were found to contribute to immune suppression and correlate with relapse in colorectal cancer (2). Domblides et al. and Kang et al. also discuss the negative impact of Tregs on functions of mature TLSs.

In conclusion, 1) “early TLSs” or “immature TLSs” exhibit distinct cellular composition and functions compared to “mature TLSs”, 2) “mature TLSs” are heterogenous and need to be defined by additional markers other than CD21+CD23+ FDC. The role of TLSs in maintaining immune niches for TCF1+PD1+ stem cells needs to be further investigated (3).

## TLS Formation Exhibits No Strict Dependence on Tumor Mutational Burden (TMB)

By analyzing diagnostic hematoxylin and eosin (H&E) images and genetic features of MIBC tumors in TCGA data, Pagliarulo et al. show that TLS density is a significant favorable prognostic factor without direct correlation with TMB. Both favorable prognosticators synergize. They propose a joint TLS-TMB score independent of tumor stage and vascular invasion. Domblides et al. also address this question and present a comprehensive analysis of the literature comparing genomic instability, oncogenic drivers, as well as viruses and TLS presence in human tumors. Presence of TLS in transcriptomic data could be assessed using the 12-chemokine (12-CK) score (4). Li et al. performed a pan-cancer comparison between the expression levels of the 12-CK score in tumors and normal samples with the TMB in tumors from TCGA. They found that 12-CK scores generally corresponded with the median tumor mutational burden (TMB) (r=0.46, p=0.01). However, mutationally silent testicular seminoma as well as several tumors with low mutational burden such as soft tissue sarcoma (5) and melanoma (6) exhibit TLSs confirming that other antigenic stimuli than TMB may be involved in TLS formation. High 12-CK scores were seen amongst cancers with high TMB, presumably with high neoantigenic stimuli to trigger a strong immunogenic response, high immune infiltrate, and presence of mature TLS, and were associated with favorable outcome for the patients. Li et al. also provide arguments supporting the use of the 12-CK score to predict response to immune checkpoint blockade. Whether the score can be refined further to take into account the TLS heterogeneity described above is an open question.

## Therapeutic Induction of TLS

The presence of intratumoral TLSs in many cancer forms are predictive of a positive response to cancer immunotherapies (Trüb and Zippelius), sparking an interest in inducing TLSs as a means to improve immunotherapy responses. Van de Walle et al. hypothesize that this may be of special benefit in CNS tumors, where TLSs could provide a local site for T cell priming. That would circumvent the need for transport of tumor antigens and trafficking of antigen-presenting cells from the CNS into cervical lymph nodes. Several papers cite the importance of vascular normalization for TLS induction in tumors (Johansson-Percival and Ganss *;* Vella et al.; Filderman et al.). STING (STimulator of INterferon Genes) agonists originally developed as anti-angiogenic agents induce immature TLSs in mice. Their use in therapy would require activation of CXCL13 production to induce TLS maturation, that could be provided upon stimulation of stromal cells (Filderman et al.). Cytokine fusion compounds which deliver TNF or IFNβ to tumor vessels, do not induce mature TLSs as monotherapies, but could be used in combination with antibodies targeting immune checkpoint molecules as immunotherapeutic tools (Johansson-Percival and Ganss). Kang et al. propose to combine the induction of HEVs and inhibition of local immunosuppression using anti-checkpoint antibodies to induce TLSs, arguing for a better knowledge of the mechanisms of regulation of HEV formation.

Domblides et al. comprehensively review the syngeneic, humanized, carcinogen-induced, and genetically engineered tumor models to induce TLSs as well as artificial approaches using organoids or spheroids. Aoyama et al. focus on approaches to artificially induce TLS formation using the LTβR-chemokine axis embedded in a variety of three-dimensional materials that are permissible to cellular infiltration and that may allow for cell-scaffold interactions. They compare the use of collagen-based matrices, hydrogels or cryogels, silica-based scaffolds, and liposome based-micro and nanoparticles with delayed release of soluble factors. Some of these materials can induce an inflammatory response due to local activation of neutrophils and macrophages, which may interfere with formation of TLSs with anti-tumor function. Aoyama et al. anticipate the challenges to translate such approaches to the clinic given the multimodal processes involved in their development.

Altogether, this Research Topic emphasizes the importance of intrinsic and cancer-dependent TLS heterogeneity, unveiling the need for their detailed and robust characterization. It also highlights the need for nomenclature adjustment in order to allow an integration of reports, and facilitate the development of new concepts and strategies for their induction.

## Author Contributions

All authors listed have made a substantial, direct, and intellectual contribution to the work and approved it for publication.

## Conflict of Interest

The authors declare that the research was conducted in the absence of any commercial or financial relationships that could be construed as a potential conflict of interest.

## Publisher’s Note

All claims expressed in this article are solely those of the authors and do not necessarily represent those of their affiliated organizations, or those of the publisher, the editors and the reviewers. Any product that may be evaluated in this article, or claim that may be made by its manufacturer, is not guaranteed or endorsed by the publisher.
